# Supplementary figures and images for: Integrative single-cell RNA-seq analysis of vascularized cerebral organoids
Source: BMC Biol. 2023 Nov 9;21:245. doi: 10.1186/s12915-023-01711-1 (PMC10634128; doi:10.1186/s12915-023-01711-1)

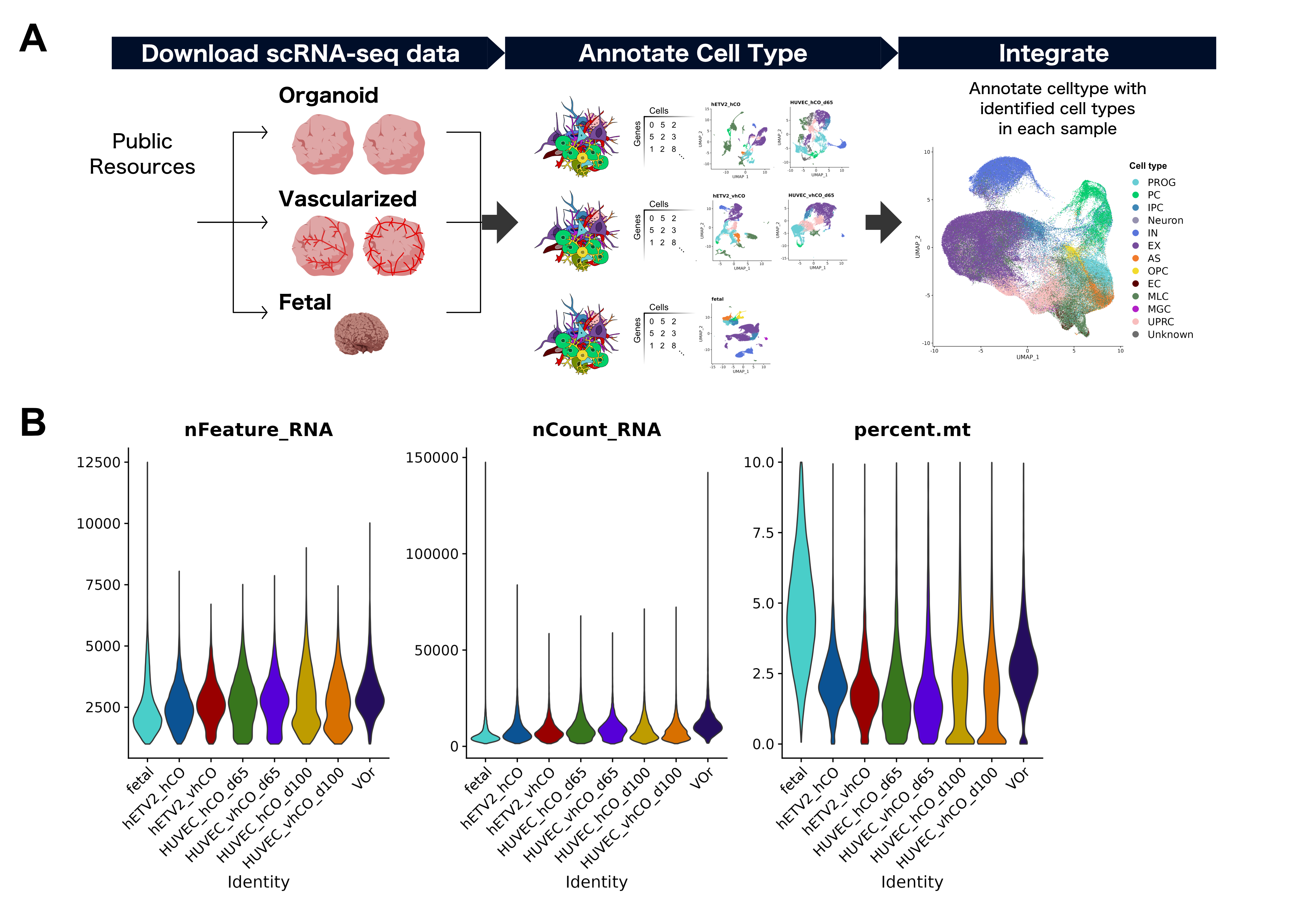

Supplement: Supplementary file 1 — Additional file 1: Fig. S1. The pre-processing of multiple scRNA-seq data. (A) Analysis method overview. Cell types were individually assigned and integrated for intravascular organoids, non-vascular organoids, and fetal brains collected from public databases. (B) Parameters for each sample. Left panel: amount of characteristic RNA expression; middle panel: total RNA expression; right panel: mitochondrial expression. [file 12915_2023_1711_MOESM1_ESM.tiff]

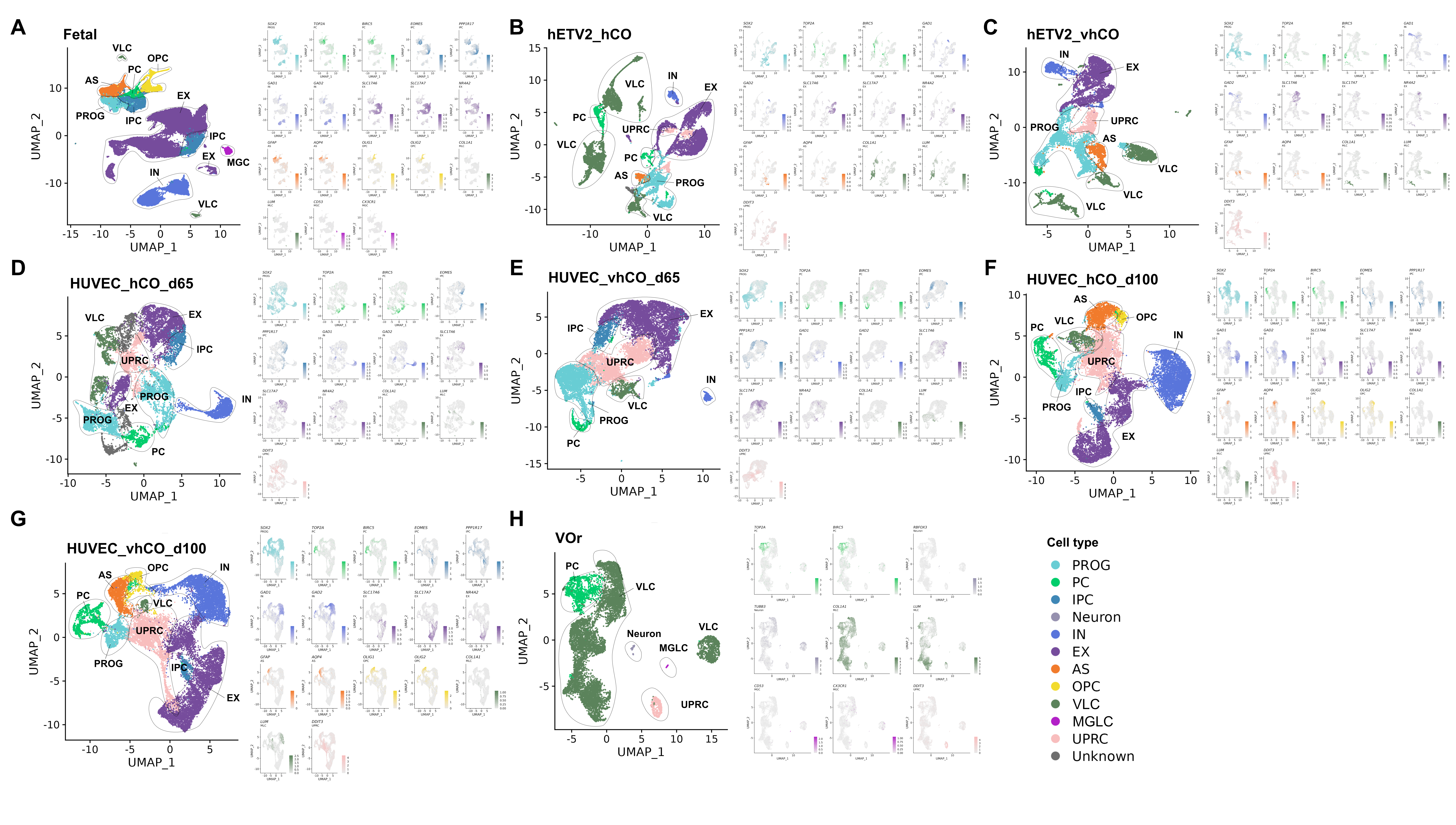

Supplement: Supplementary file 2 — Additional file 2: Fig. S2. Characteristic gene expression profiles of annotated cell types. (A-H) Expression of marker genes corresponding to cell types in each sample. [file 12915_2023_1711_MOESM2_ESM.tiff]

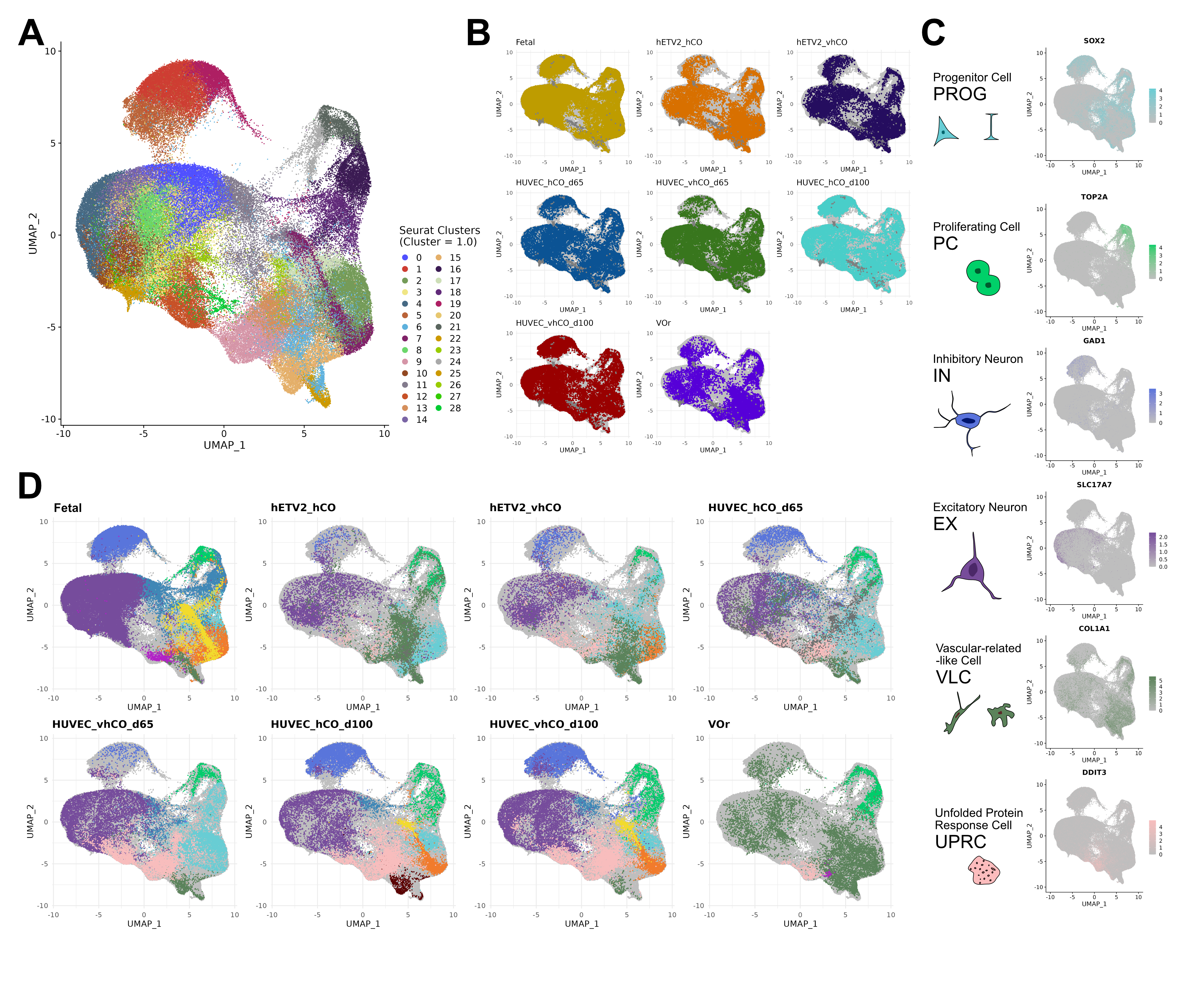

Supplement: Supplementary file 3 — Additional file 3: Fig. S3. Integration of scRNA-seq data. (A) Clusters of integrated samples. (B) UMAP for each integrated sample. (C) UMAP indicating cell types in each integrated sample. (D) Expression of each marker gene. [file 12915_2023_1711_MOESM3_ESM.tiff]

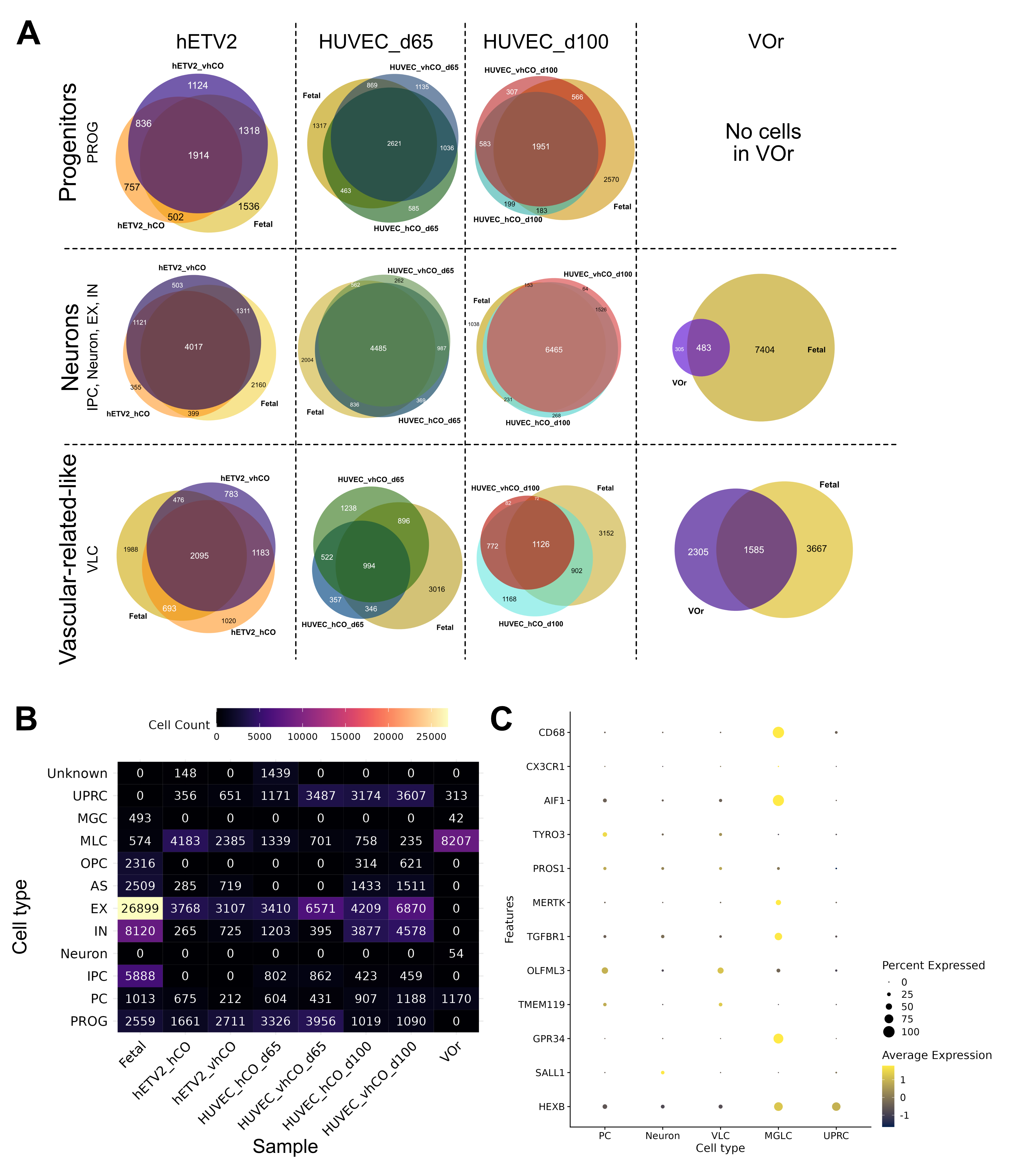

Supplement: Supplementary file 5 — Additional file 5: Fig. S5. scRNA-seq data analysis in ectoderm cell subtypes. (A) Overlap of characteristic genes for each cell type between each organoid and corresponding to the fetal brain. Differentially expressed genes specific to each cell group were identified with a cut-off at corrected p-value. The genes overlapping with the Venn diagram were plotted using the “ggvenn” package (v0.1.9). (B) Cell counts for each cell type in each sample. (C) Dot plot of expression of microglial markers in VOr samples. [file 12915_2023_1711_MOESM5_ESM.tiff]

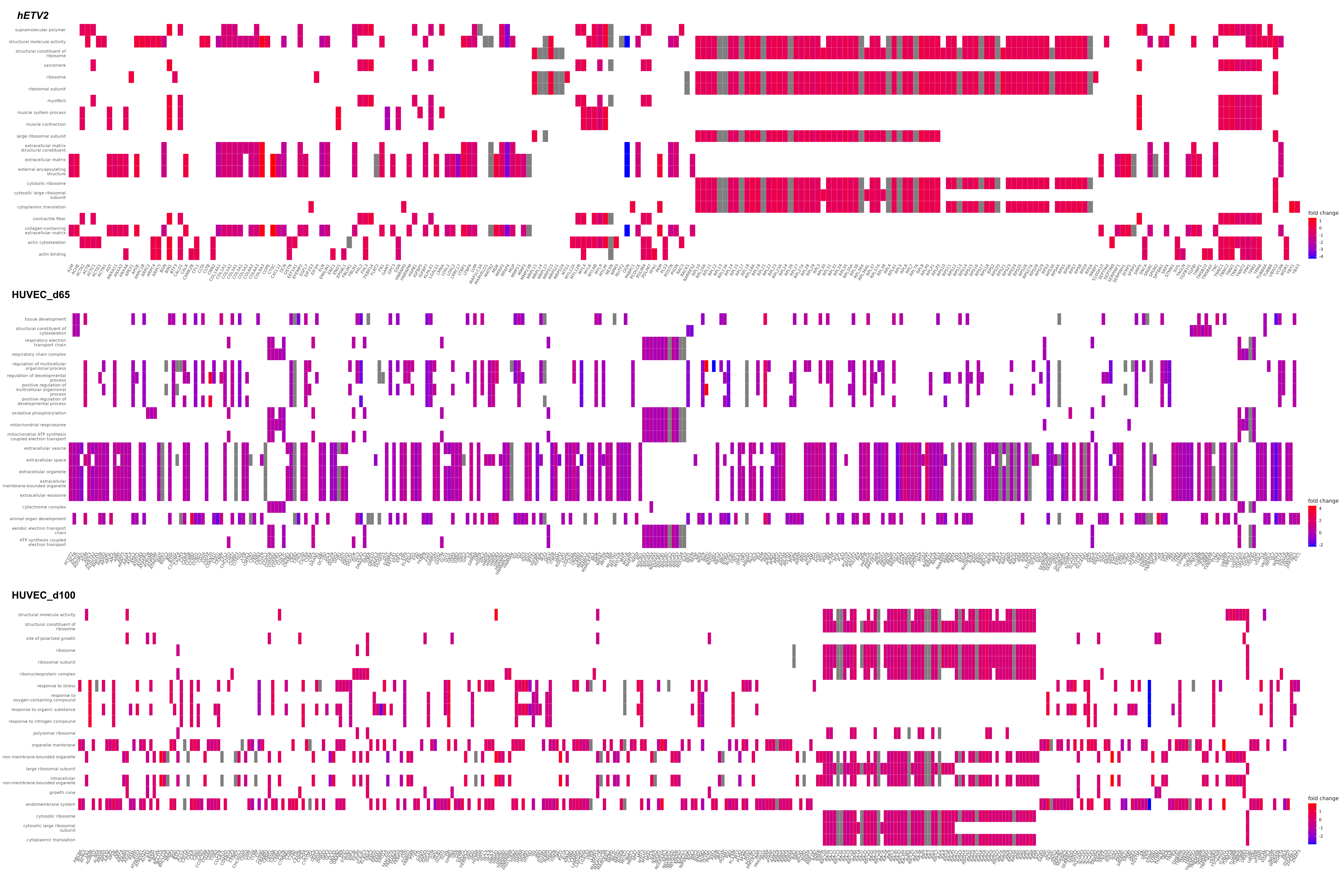

Supplement: Supplementary file 7 — Additional file 7: Fig. S6. GO terms altered by vascularization. Plot of the differential expression levels of the gene sets characteristic of each GO term, visualized by the “heatplot” function of the “enrichplot” package (v1.16.2). The “ENTREZID” were converted to gene symbols using the “setReadable” function of the “DOSE” package (v3.22.1). [file 12915_2023_1711_MOESM7_ESM.tiff]

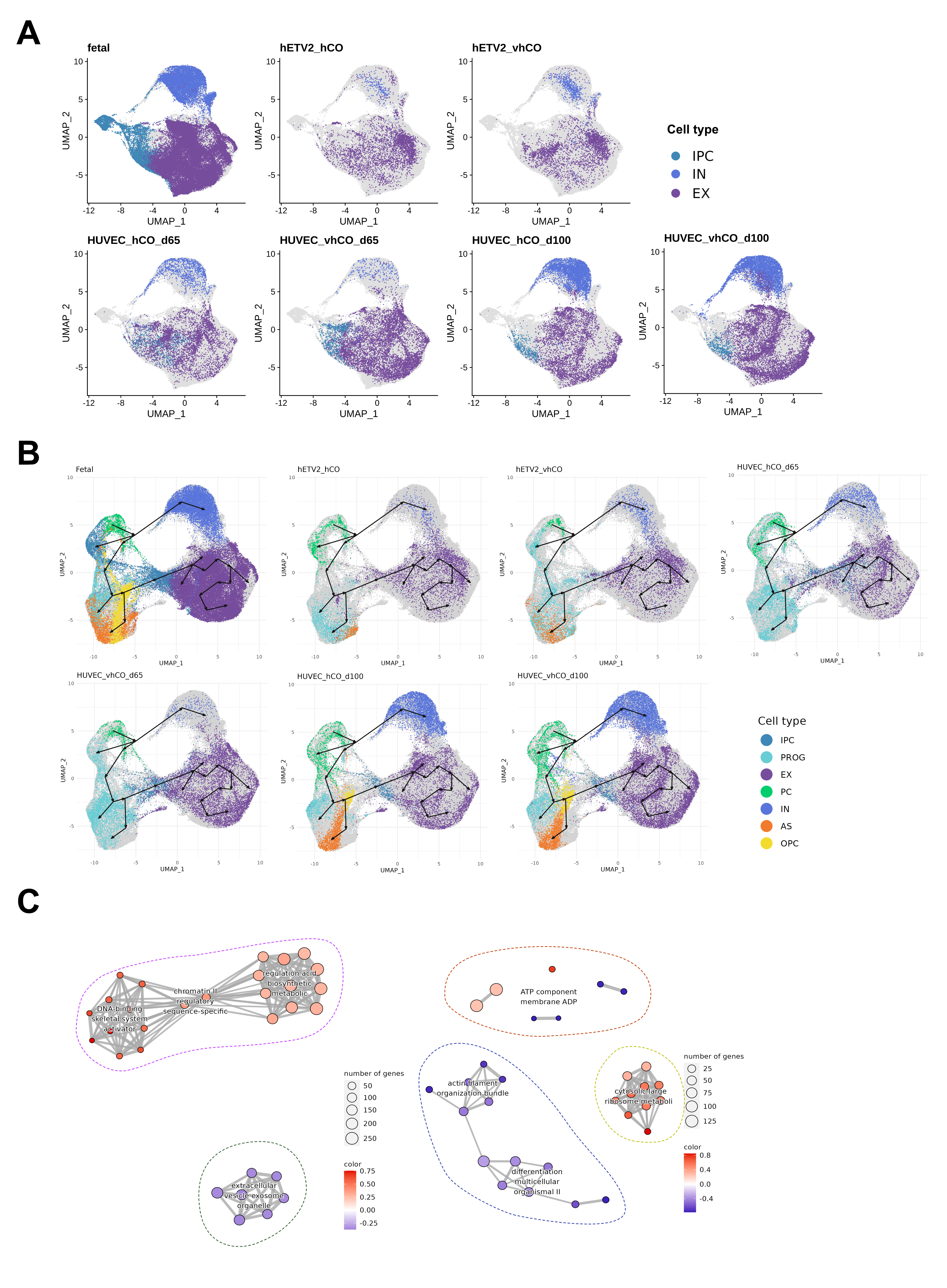

Supplement: Supplementary file 9 — Additional file 9: Fig. S7. Integration and downstream analysis of neuronal subtypes. (A) UMAP in each sample of extracted neurons (IPC, EX, IN). (B) Differentiation trajectories of ectodermal cells revealed by trajectory analysis. (C) Alterations in GO-terms induced by vascularization in neurons. Note that color indicates enrichment score, not p-value. [file 12915_2023_1711_MOESM9_ESM.tiff]

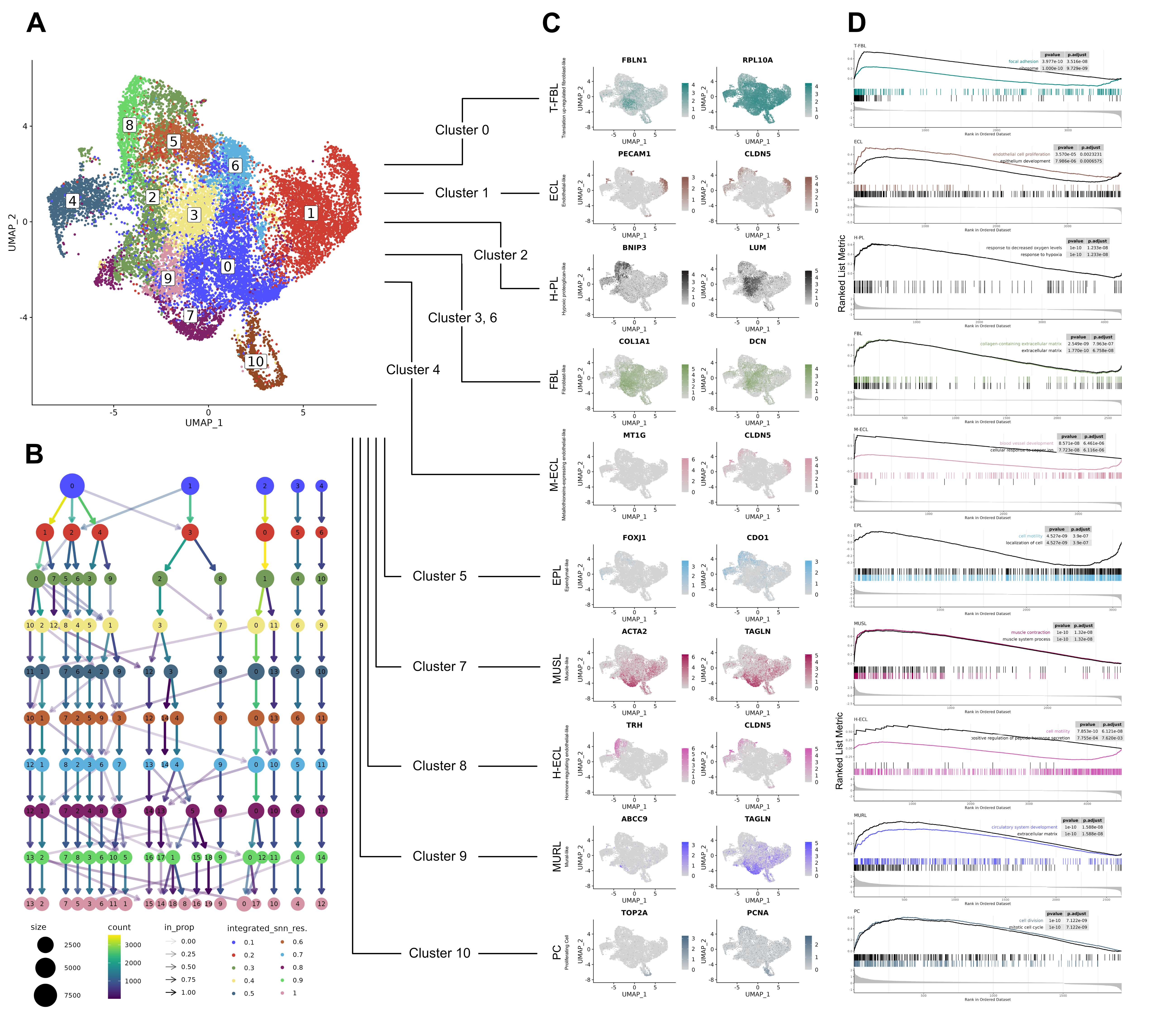

Supplement: Supplementary file 11 — Additional file 11: Fig. S8. Evidence for determination of VLC subtypes. (A) Eleven clusters of resolution = 0.3 obtained unbiased. The resolution was determined by the “clustree” shown in S8B Fig. For each cluster, a cell type was assigned based on the evidence in S8C and S8D Fig. (B) Clustree plot representing cluster relationships at resolutions from 0.1 to 1 (step 0.1) using the “clustree” package (v0.5.0). The dot size indicates the number of cells, and the line extending from the cluster indicates the cluster relationship. (C) Expression levels of genes that were characteristically expressed in each cluster. The color of the plots matches the concept color of each cell type. (D) GSEA results calculated based on non-biased computed gene sets. The table on the right side presents the p-value for each GO term. The plots were generated by the “gseaplot2” function of the “enrichplot” package. [file 12915_2023_1711_MOESM11_ESM.tiff]

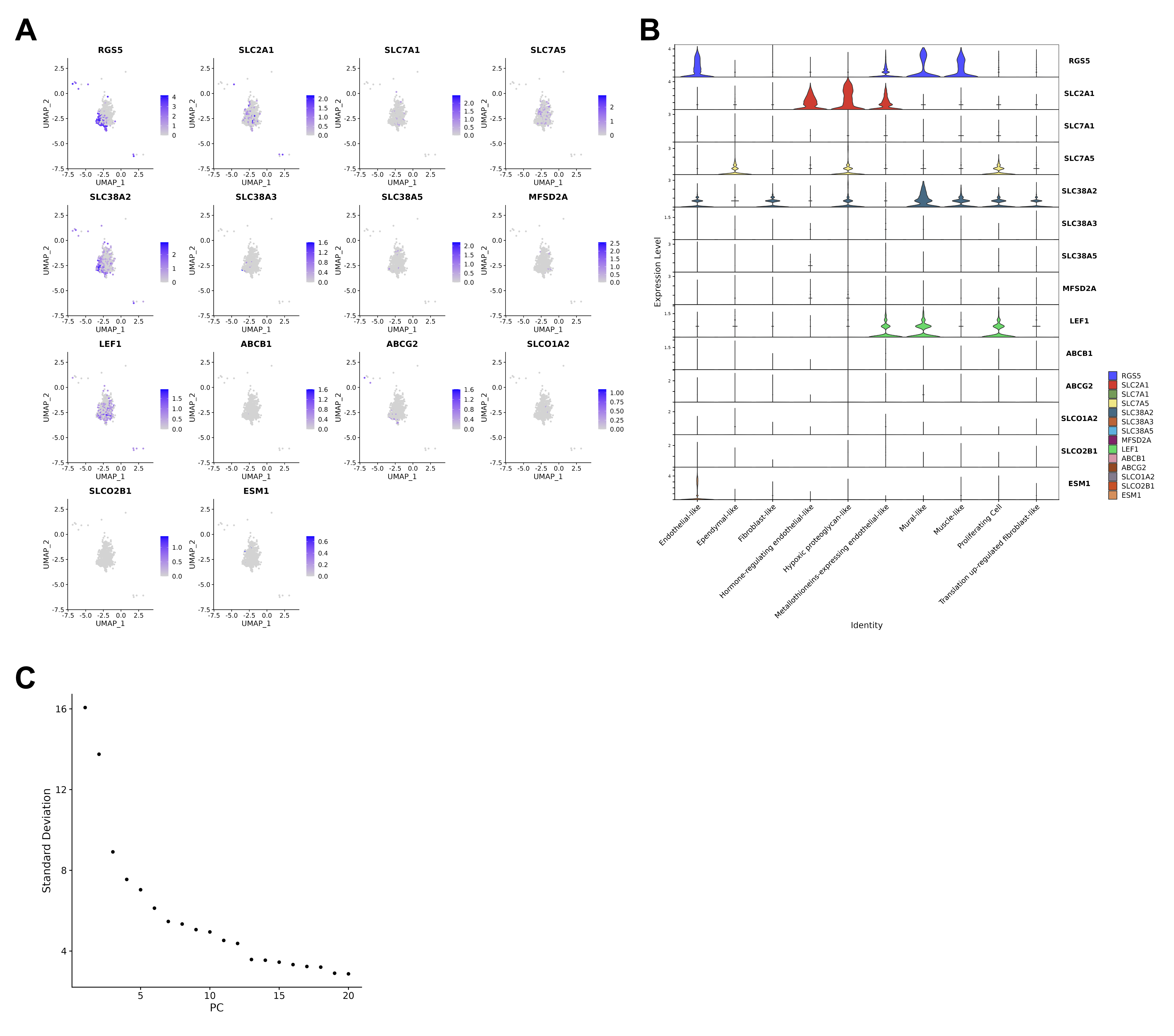

Supplement: Supplementary file 14 — Additional file 14: Fig. S9. BBB-related genes and cluster determination. (A) Feature plot of BBB-related genes expression in pericyte-like clusters. (B) Violin plot of BBB-related genes expression in pericyte-like clusters. (C) Plot of standard deviation for each feature dimension to select the feature dimension with the smallest change in standard deviation. [file 12915_2023_1711_MOESM14_ESM.tiff]
